# Genetic correlation analysis between sepsis and hematological traits: Identifying shared genomic regions

**DOI:** 10.1371/journal.pone.0333675

**Published:** 2025-11-14

**Authors:** Jianmin Zhang, Miao Hou, Zhenjiang Bai, Jian Wang, Jie Huang

**Affiliations:** 1 Department of Traditional Chinese Medicine, Children’s Hospital of Soochow University, Suzhou, Jiangsu, China; 2 Department of Cardiology, Children’s Hospital of Soochow University, Suzhou, Jiangsu, China; 3 Department of Critical Care Medicine, Children’s Hospital of Soochow University, Suzhou, Jiangsu, China; 4 Department of Surgery, Children’s Hospital of Soochow University, Suzhou, Jiangsu, China; The Ohio State University, UNITED STATES OF AMERICA

## Abstract

**Background:**

Sepsis, dysregulated cascades of inflammatory response to infection, remains a critical clinical condition, leading to morbidity and mortality. Better understanding of the genetic basis underlying sepsis has the potential to better prevent and treat this potentially life-threatening condition. Recent studies have identified genetic variants for sepsis and accumulated evidence for the relevance of hematological traits. However, genetic correlation analysis, testing the hypothesis of shared genetic underpinning of sepsis and hematological traits, as well as identifying shared specific genetic regions, is lacking.

**Method:**

In this study, we systematically evaluated the extent and statistical significance of global genetic correlation between sepsis and a comprehensive battery of 29 hematological traits, as well as performed local genetic correlation analysis to pinpoint the shared genomic regions.

**Results:**

Our analyses revealed significant global genetic correlation between sepsis and five red blood cell indices. Local genetic correlation analyses identified 6–21 genomic regions with lengths ranging from 1.4kb to 5.6Mb shared between sepsis and seven hematological traits.

**Conclusion:**

Our findings improve our general understanding of the shared genetics between sepsis and different categories of blood cell traits, and have the potential to advance personalized medicine for sepsis.

## Introduction

Sepsis is a life-threatening organ dysfunction triggered by a cascade of dysregulated and overwhelming host responses to an infection. Sepsis is often associated with widespread inflammation, tissue damage, organ failure, and even death [1–3]. Sepsis unfortunately affects people of any age group: for example, pediatric sepsis afflicting children under one year of age; while among adults literature has reported increased risk for people above 65 years old. Immune-compromised individuals and people with pre-existing and/or chronic medical conditions are also at elevated risk of developing sepsis [4]. According to the 2024 Centers for Disease Control and Prevention estimates [5], each year at least 1.7 million US adults become septic; at least 350,000 die because of it.

The incidence of sepsis has been increasing, and the disease has been frequently associated with chronic physical, psychological, and cognitive impairments, as well as much reduced quality of life [6]. The mortality rate for sepsis was reported to be as high as 35–45% and is still at 18.4% [7]. Investigators have been making efforts to decipher the mechanisms behind this disease for better diagnosis, prevention and treatment. The etiopathology underlying sepsis has been recognized as complicated and obscure, both in terms of disease development and progress to its severe forms.

Immune response to infection in general and sepsis have both been reported to be at least partially genetically determined [8,9]. Genetic association studies have identified multiple loci associated with sepsis [8–11] and the polygenic risk scores constructed have been reported to show promising discriminating power between people affected with sepsis and controls [8].

Despite the advancement of genetic variants identified for sepsis and its close connection to immune response, genetic correlation between sepsis and immune biomarkers has been under-explored. More importantly, published studies investigating genetic correlation between sepsis and other related diseases and traits [12,13] focus on global genetic correlation, failing neither to reflect the complexity of shared genetic architecture between sepsis and other phenotypes nor to identify genetic regions contributing to the global genetic correlation. In this study, we attempt to fill the gaps by both comprehensively estimating the global genetic correlation between sepsis and the battery of hematological biomarkers from routine complete blood cell count (CBC) tests and pinpointing genetic regions where the global genetic correlations can be attributed to.

## Methods

### Overview

**Fig 1:**
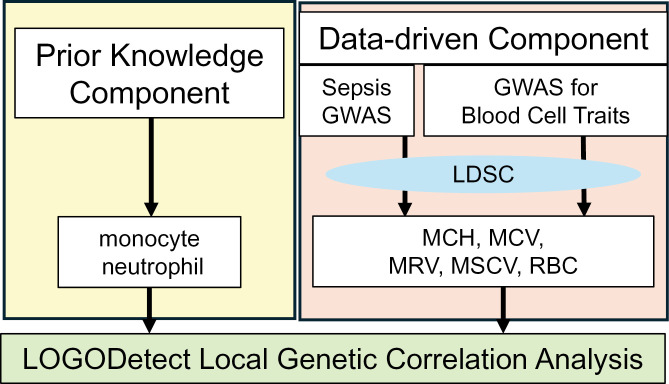
Overall study design. We selected blood cell traits that are potentially relevant to sepsis through two sources: based on prior knowledge where monocyte and neutrophil counts are selected (top left box with yellow color background); and based on data-driven global correlation analysis using LD score regression (LDSC) based on sepsis and blood cell traits GWAS results, which leads to five additional traits including MCH, MCV, MRV, MSCV and RBC (top right box with peach color background). Combined together, the resulting seven blood cell traits are further subject to local genetic correlation analysis using LOGODetect with sepsis. MCH: mean corpuscular hemoglobin; MCV: mean corpuscular volume; MRV: mean reticulocyte volume; MSCV: mean sphered corpuscular volume; RBC: red blood cell count.

Our main objective is to identify local genetic regions that account for the shared genetic underpinning of sepsis and hematological traits. In order to detect these genetic regions, we performed local genetic correlation analysis based on findings from genome-wide association studies (GWAS) for sepsis and each of the relevant hematological traits. There is a comprehensive battery of blood cell traits, including white blood cell indices, red blood cell traits, and platelet indices, for which genetic association studies have been performed in large samples of individuals from multiple population groups [14–16]. To select the relevant blood cell traits, we adopted a two-component approach (**Fig 1**) with one component based on prior knowledge from literature, and the other component driven by data. For the second component, specifically, we carry out global genetic correlation, using LDSC [17], to select hematological traits that are genetically correlated with sepsis.

### GWAS summary statistics

There are multiple published GWAS studies for sepsis [10,11,18], also including survival outcome [19], pediatric sepsis [20]. Similarly there are many published GWAS for blood cell indices [14–16,21,22]. Considering multiple factors including sample size, genome coverage of genetic variants interrogated, sample ascertainment (particularly for sepsis where most of the large-sample size studies are based on biobank data with sepsis cases defined with suboptimal ICD9 or ICD10 codes), comprehensiveness of hematological traits analyzed, and availability of genome-wide summary statistics, we chose results from Vuckovic et al. [16] for blood cell traits and summary statistics from Butler-Laporte et al. [11] for subsequent analyses.

### Global genetic correlation analysis

Global genetic correlation analysis was performed using the LD Score Regression (LDSC) method [17,23]. GWAS summary statistics from Butler-Laporte et al. [11] and Vuckovic et al. [16] were used to estimate global genetic correlation as well as to test the statistical significance of the genetic correlation between sepsis and each of the following 29 blood cell phenotypes: four platelet related traits including platelet count (PLT), mean platelet volume (MPV), platelet distribution width (PDW), and plateletcrit (PCT); 8 indices pertinent to mature red blood cells (including red blood cell count (RBC), mean corpuscular volume (MCV), hematocrit (HCT), mean corpuscular hemoglobin (MCH), mean sphered corpuscular volume (MSCV), mean corpuscular hemoglobin concentration (MCHC), hemoglobin concentration (HGB), red cell distribution width (RDW)), 6 indices pertinent to immature red blood cells including reticulocyte count (RET), reticulocyte fraction of red cells (RET%), immature fraction of reticulocytes (IRF), high light scatter reticulocyte count (HLR), High light scatter reticulocyte percentage of red cells (HLR%), and mean reticulocyte volume (MRV); four traits for myeloid white blood cell counts including monocyte count (MONO), neutrophil count (NEUT), eosinophil count (ESO), basophil count (BASO); lymphocyte count (LYMPH); total white blood cell count (WBC); and five percentages of white blood cell subtypes including monocyte percentage (MONO%), neutrophil percentage (NEUT%), eosinophil percentage (ESO%), basophil percentage (BASO%), and lymphocyte percentage (LYMPH%).

### Local genetic correlation analysis

To pinpoint genomic regions harboring shared genetic underpinnings behind sepsis and hematological indices, we carried out local genetic correlation analysis using GWAS summary statistics for sepsis [11] and for each of the 29 blood cell phenotypes [16]. Specifically, we employed the LOGODetect method [24] to perform local genetic correlation analysis between sepsis and the 29 blood cell indices detailed in the “Global genetic correlation analysis” section above.

## Results

### GWAS summary statistics

GWAS summary statistics from Vuckovic et al. [16] for a battery of 29 blood cell indices were derived from 408,112 European ancestry individuals. The number of variants examined for association analysis is around 41 million, ranging from 41,266,573 for reticulocyte fraction of red cells to 41266573 for lymphocyte count. The number of significant variants exceeding genome-wide significance threshold of 5E-8 ranges from 17,358 for basophil percentage of white cells to 174,249 for mean sphered corpuscular volume. In contrast, there were no genome-wide significant variants for sepsis, despite the large sample size of 18,931 cases and 663,531 controls.

### LDSC analysis reveals five red blood cell indices genetically correlated with sepsis

Our global genetic correlation analysis using LDSC (summarized in **Table 1**) identified nominally significant (LDSC *P* value < 0.05) correlation between sepsis and multiple blood cell phenotypes, including mean corpuscular hemoglobin (MCH) (genetic correlation *r*_*g*_ = 0.1286, *P* value = 0.0259), mean corpuscular volume (MCV) (*r*_*g*_ = 0.142, *P* value = 0.0094), mean reticulocyte volume (MRV) (*r*_*g*_ = 0.1181, *P* value = 0.0127), mean sphered corpuscular volume (MSCV) (*r*_*g*_ = 0.1242, *P* value = 0.0055), and red blood cell count (RBC) (*r*_*g*_ = −0.1084, *P* value = 0.0256). Interestingly, all of these significant hematological traits are pertinent to red blood cells, both with mature red blood cells (MCH, MCV, MSCV, and RBD) or with immature red cells (MRV). No significant genetic correlation was detected between sepsis and white blood cell indices including neutrophils or monocytes that have been linked to sepsis in the literature. The results can be partially driven by the higher heritability of the red blood cell phenotypes. For example, MSCV showing the most significant genetic correlation with sepsis (LDSC *P* value = 0.0055) has the highest heritability estimate among the five red blood cell indices that exhibit significant genetic correlation. As a matter of fact, heritability estimates for all of the five indices rank among the top 8, ranging from 0.2307 to 0.2881 (**Table 1**). In comparison, neutrophil and monocyte indices, including counts and percentages, all have lower heritability estimates, with the highest of 0.2252 for monocyte count, and all the other three much lower ranging from 0.1534 to 0.1819.

**Table 1:** Global genetic correlation results using LDSCLDSC.

Cell Type	BCT^*^ abbreviation	BCT Full Name	rg^#^	se^^^	z	p	h2^&^	h2 se^^^
**Platelet**	**PLT**	Platelet count	-−0.0319	0.0446	-−0.7158	0.4741	0.2798	0.0218
	**MPV**	Mean platelet volume	-−0.0434	0.0469	-−0.9257	0.3546	0.2937	0.0307
	**PDW**	Platelet distribution width	0.0405	0.0796	0.5085	0.6111	0.2072	0.022
	**PCT**	Plateletcrit	-−0.0691	0.044	-−1.5699	0.1164	0.2431	0.0187
**Mature red cell**	**RBC**	Red blood cell count	-−0.1084	0.0486	-−2.2323	0.0256	0.2309	0.0185
	**MCV**	Mean corpuscular volume	0.142	0.0547	2.5974	0.0094	0.2755	0.0254
	**HCT**	Hematocrit	-−0.0308	0.0497	-−0.6193	0.5357	0.1733	0.0129
	**MCH**	Mean corpuscular hemoglobin	0.1286	0.0577	2.2282	0.0259	0.2507	0.0252
	**MSCV**	Mean sphered corpuscular volume	0.1242	0.0447	2.7746	0.0055	0.2881	0.0237
	**MCHC**	Mean corpuscular hemoglobin concentration	-−0.0225	0.0541	-−0.4152	0.678	0.0931	0.0099
	**HGB**	Hemoglobin concentration	-−0.0426	0.0503	-−0.8454	0.3979	0.1755	0.0126
	**RDW**	Red cell distribution width	0.0759	0.0659	1.1508	0.2498	0.2213	0.0193
**Immature red cell**	**RET**	Reticulocyte count	-−0.0309	0.0501	-−0.6162	0.5378	0.2077	0.0187
	**RET%**	Reticulocyte fraction of red cells	-−0.0062	0.0544	-−0.1135	0.9096	0.2015	0.0186
	**IRF**	Immature fraction of reticulocytes	0.0531	0.0559	0.95	0.3421	0.1314	0.0124
	**HLR**	High light scatter reticulocyte count	-−0.0053	0.052	-−0.1028	0.9181	0.2093	0.0185
	**HLR%**	High light scatter reticulocyte percentage of red cells	0.0116	0.055	0.211	0.8329	0.2058	0.0185
	**MRV**	Mean reticulocyte volume	0.1181	0.0474	2.4912	0.0127	0.2307	0.0203
**Myeloid white cell**	**MONO**	Monocyte count	-−0.0231	0.0542	-−0.4259	0.6702	0.2252	0.0231
	**NEUT**	Neutrophil count	-−0.0583	0.0657	-−0.8881	0.3745	0.1702	0.0121
	**ESO**	Eosinophil count	0.0312	0.0572	0.5458	0.5852	0.2062	0.0209
	**BASO**	Basophil count	-−0.0667	0.078	-−0.8543	0.393	0.0704	0.0068
**Lymphoid white cell**	**LYMPH**	Lymphocyte count	-−0.0277	0.0478	-−0.5793	0.5624	0.2253	0.0161
**Compound white cell**	**WBC**	White blood cell count	-−0.0476	0.06	-−0.7935	0.4275	0.2071	0.0132
	**MONO%**	Monocyte percentage of white cells	0.0083	0.0517	0.1602	0.8728	0.1819	0.0207
	**NEUT%**	Neutrophil percentage of white cells	-−0.0475	0.0566	-−0.8393	0.4013	0.1534	0.0111
	**ESO%**	Eosinophil percentage of white cells	0.059	0.0621	0.9488	0.3427	0.1961	0.0202
	**BASO%**	Basophil percentage of white cells	-−0.0347	0.0705	-−0.4925	0.6223	0.0539	0.0061
	**LYMPH%**	Lymphocyte percentage of white cells	0.0313	0.0525	0.5961	0.5511	0.1694	0.0128

* BCT: Blood cell trait

^#^ rg: (estimated) global genetic correlation

^ se: standard error

^&^ h^2^: (estimated) heritability

### LOGODetect narrows down genomic regions shared behind sepsis and blood cell traits

As illustrated in **Fig 1**, we performed local genetic correlation analysis using the LOGODetect method for the five blood cell phenotypes that show significant global genetic correlation with sepsis (namely MCH, MCV, MSCV, MRV, and RBD), as well as for the monocyte and neutrophil counts whose relevance to sepsis has been reported by previous studies.

**Fig 2:**
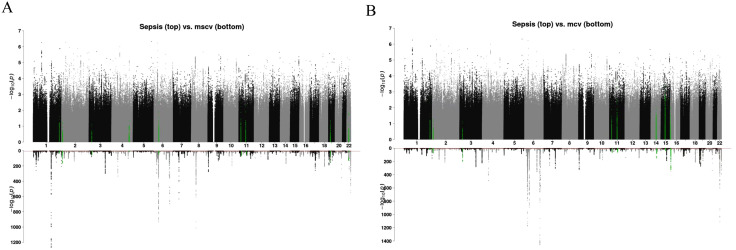
Local genetic correlation results for selected red blood cell indices. Local genetic correlation results for two selected red blood cell indices are visualized through mirrored Manhattan plots. There are two sub-figures, for MSCV (A) and MCV (B) respectively. For each sub-figure, we show a mirrored Manhattan plot where the top panel shows the -log10(p-value) of sepsis GWAS on the top panel and the -log10(p-value) of the red blood cell trait GWAS on the bottom panel.

LOGODetect identified 6−21 genomic regions, for the seven tested blood cell phenotypes, with lengths ranging from 1.4kb to 5.6Mb that are locally correlated with sepsis genetics. The results are summarized in S1 File. **Fig 2** visualizes results for MSCV that showed the most significant genetic correlation (*P* value = 0.0055) and for MCV that showed the largest magnitude of genetic correlation (*r*_*g*_ = 0.142) based on LDSC analyses presented above. We observed partial overlaps between the two groups of identified regions. Among the 8 regions identified between MCV and sepsis, and the 9 regions between MSCV and sepsis, four regions (chr1: 229,737,680–229,802,996, chr2: 8,718,430–8,775,344, chr3: 24,340,817–24,357,286, and chr11: 19,208,070–19,277,199) are shared, reflecting shared genetic underpinnings behind all three of the phenotypes. As a matter of fact, the chr11: 19,208,070–19,277,199 region was identified also for MCH, MRV, and RBC (S2 File).

**Fig 3:**
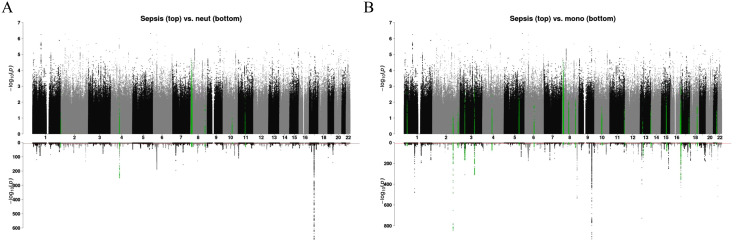
Local genetic correlation results for white blood cell indices. Local genetic correlation results for two white blood cell indices are visualized through mirrored Manhattan plots. There are two sub-figures, for neutrophil count (neut) and monocyte count (mono) respectively. For each sub-figure, we show a mirrored Manhattan plot where the top panel shows the -log10(p-value) of sepsis GWAS on the top panel and the -log10(p-value) of the white blood cell trait GWAS on the bottom panel.

**Fig 3** shows LOGODetect results between monocyte count and sepsis, and between neutrophil count and sepsis. Despite no significant global genetic correlation identified by LDSC between monocyte count and sepsis (*r*_*g*_ = −0.0231, *P* value = 0.6702), the largest number of genomic regions (21 compared to ≤ 15 for other blood cell traits subjected to LOGODetect analysis) were revealed from local genetic correlation analysis. More interestingly, none of the 21 regions overlapped with the regions identified for the other six blood cell traits, suggesting that genetic contributions shared between monocyte count and sepsis are largely independent of the other blood cell indices analyzed.

## Discussion

Our study is the first to perform systematic genetic correlation analysis between sepsis and blood cell phenotypes. Our interrogations encompass both global genetic correlation estimates and local genetic correlation analysis, the latter of which enables pinpointing specific genomic regions that may account for the shared genetic underpinning of sepsis and the corresponding hematological traits that demonstrate significant local genetic correlation in these regions.

Despite accumulated evidence in the literature supporting the relevance to sepsis of leucocytes and its subtypes, particularly monocytes, neutrophils, and lymphocytes, global genetic correlation analyses revealed significant genetic correlations only with red blood cell indices. Interestingly, despite insignificant global genetic correlation between sepsis and monocyte count, we observed the largest number of genomic regions exhibiting significant local genetic correlation. This apparent discrepancy has been observed in the literature, including several examples (such as between type 1 diabetes and lupus, asthma and hypothyroidism) highlighted in Werme et al [25], where multiple genetic regions exhibited significant local correlation despite close to zero global genetic correlation estimates. Similarly, the LOGODetect publication identified more regions for schizophrenia and ID (53 regions, *r*_*g*_ = −0.23) than for major depressive disorder and neuroticism (40 regions, *r*_*g*_ = 0.78) despite the much larger absolute correlation of the latter. The main reason, as pointed out in Werme et al [25] is that global correlation fails to capture heterogeneous genetic relations across the genome.

The lack of significant global genetic correlation is also likely a power issue that is influenced by multiple factors including the heritability both the blood cell traits (as speculated earlier in the Results section) and sepsis, and the sample sizes of the underlying GWAS studies. We note that there are no GWAS findings exceeding the stringent genome-wide significance threshold of p-value 5 × 10^−8^. Despite the large sample size (total N = 682,462) and the relatively large number of sepsis cases (7,463 from FinnGen and 11,468 from UK Biobank) in the GWAS study [11], many factors can still lead to lack of genome-wide significant signals. These factors include the possible highly polygenic genetic architecture with many variants exerting only small effects, disease heterogeneity and differential effects of genetic variants on different subtypes (which combined can lead to power loss when lumping all cases together), and common issues for multi-biobank meta-analysis including population stratification, uncontrolled environmental effects, and differential disease definitions, recruitment strategies, and participant baseline characteristics. Therefore, we strongly advocate data sharing wheneve possible and establishment of global efforts to apply best practices starting at the design stage to ensure most powerful studies, similar to the Global Biobank Meta-analysis Initiative [26]. We have made our best efforts to use the largest sample size GWAS studies to maximally mitigate the issues. Future studies revisiting genetic correlations using updated GWAS results will be valuable to further investigate the shared genetic determinants between blood cell traits and sepsis.

In addition to the power and sample size, study populations can also impact our findings. For example, different disease subtypes (adult versus pediatric, sepsis with different clinical symptoms and/or co-morbidities), may have different genetic underpinnings behind. For another example, different ancestral populations may also have distinct genetic mechanisms. In this study, we focus on genetic correlation among European ancestry individuals due to various practical considerations including sample size; the availability of large-scale GWAS (limited, if any, comparable large-scale GWAS were conducted in non-European ancestry populations, particularly for sepsis), and matching of ancestry for sepsis and blood cell traits GWAS (it is much more challenging to match for non-European ancestry populations, e.g., due to multi-ancestry meta-analyses dominated by European ancestry samples rather than broken down by distinct ancestry groups). When GWAS studies from comparable sample sizes from other population groups become available, it is worthy to further investigate across various different populations.

Despite the limitations due to practical constraints, ours represent the first systematic genetic correlation study between sepsis and the whole battery of 29 blood cell traits. Our findings advance our general understanding of the shared genetics between sepsis and different categories of blood cell traits, and reveal biological targets that have the potential to be used as drug targets for the development pharmacotherapies, and that can be personalized based on the complete blood count lab tests readily and commonly performed in clinical practice.

## Supporting information

S1 File Supplementary Table. Local genetic correlation analysis results between sepsis and 7 blood cell traits are tabulated. For each blood cell trait, all the genomic regions that exceed FDR 5% (qval < 0.05) are shown, tabulating chromosome (chr), beginning position (begin_pos), stopping position (stop_pos), LOGODetect test statistics (stat), raw p-value (pval), and multiple testing adjusted p-value (qval).
(XLSX)

S2 File Supplementary Figure. Local genetic correlation results for three additional red blood cell indices are visualized through mirrored Manhattan plots. There are three sub-figures, in the order of MCH, MRV, and RBC respectively. For each sub-figure, we show a mirrored Manhattan plot where the top panel shows the -log10(p-value) of sepsis GWAS on the top panel and the -log10(p-value) of the blood cell trait GWAS on the bottom panel.
(DOCX)
