# Supplementary material for: Genetic correlation analysis between sepsis and hematological traits: Identifying shared genomic regions
Source: PLoS One. 2025 Nov 14;20(11):e0333675. doi: 10.1371/journal.pone.0333675 (PMC12617866; doi:10.1371/journal.pone.0333675)

## Supplementary Figure. Local genetic correlation results for three additional red blood cell indices


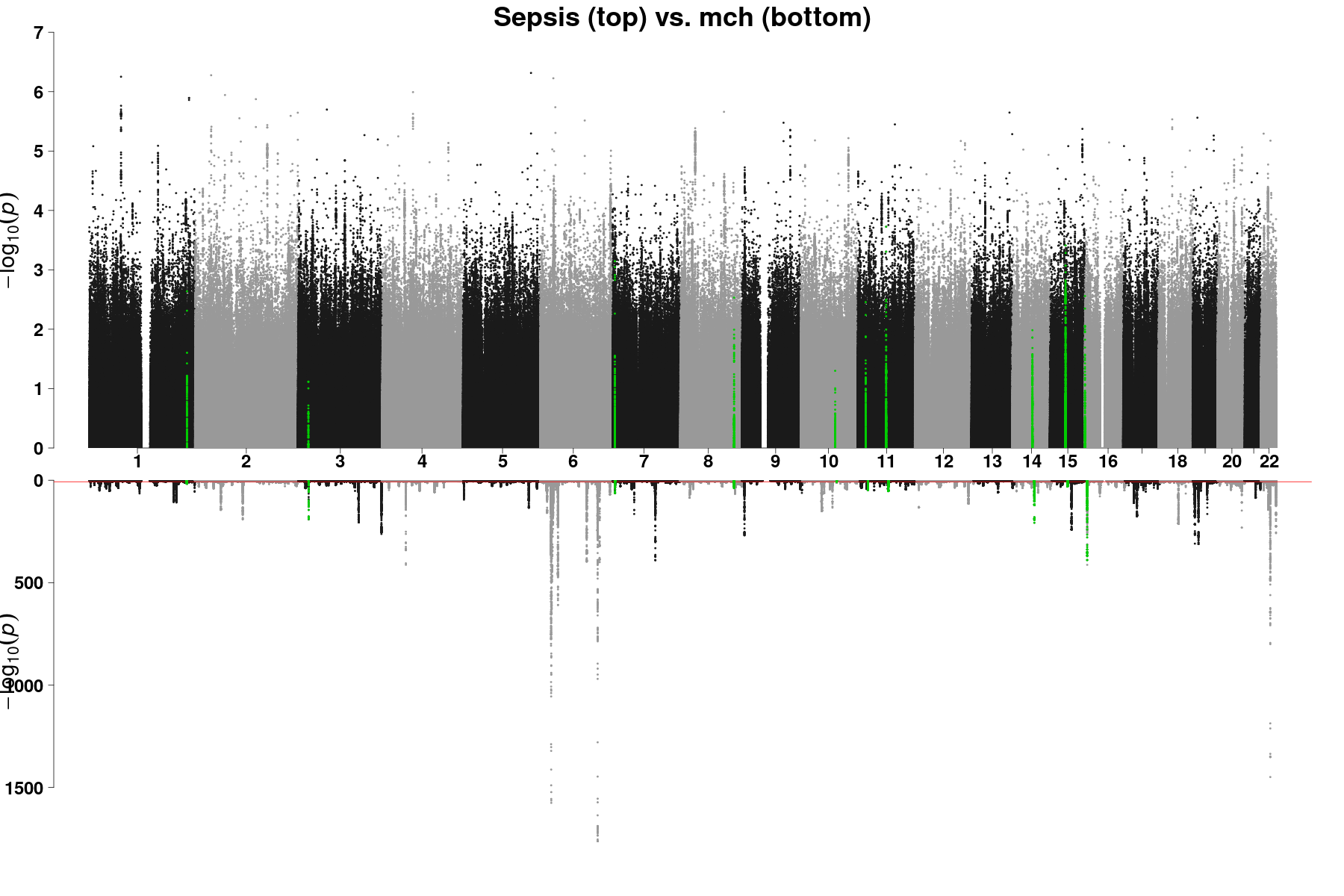


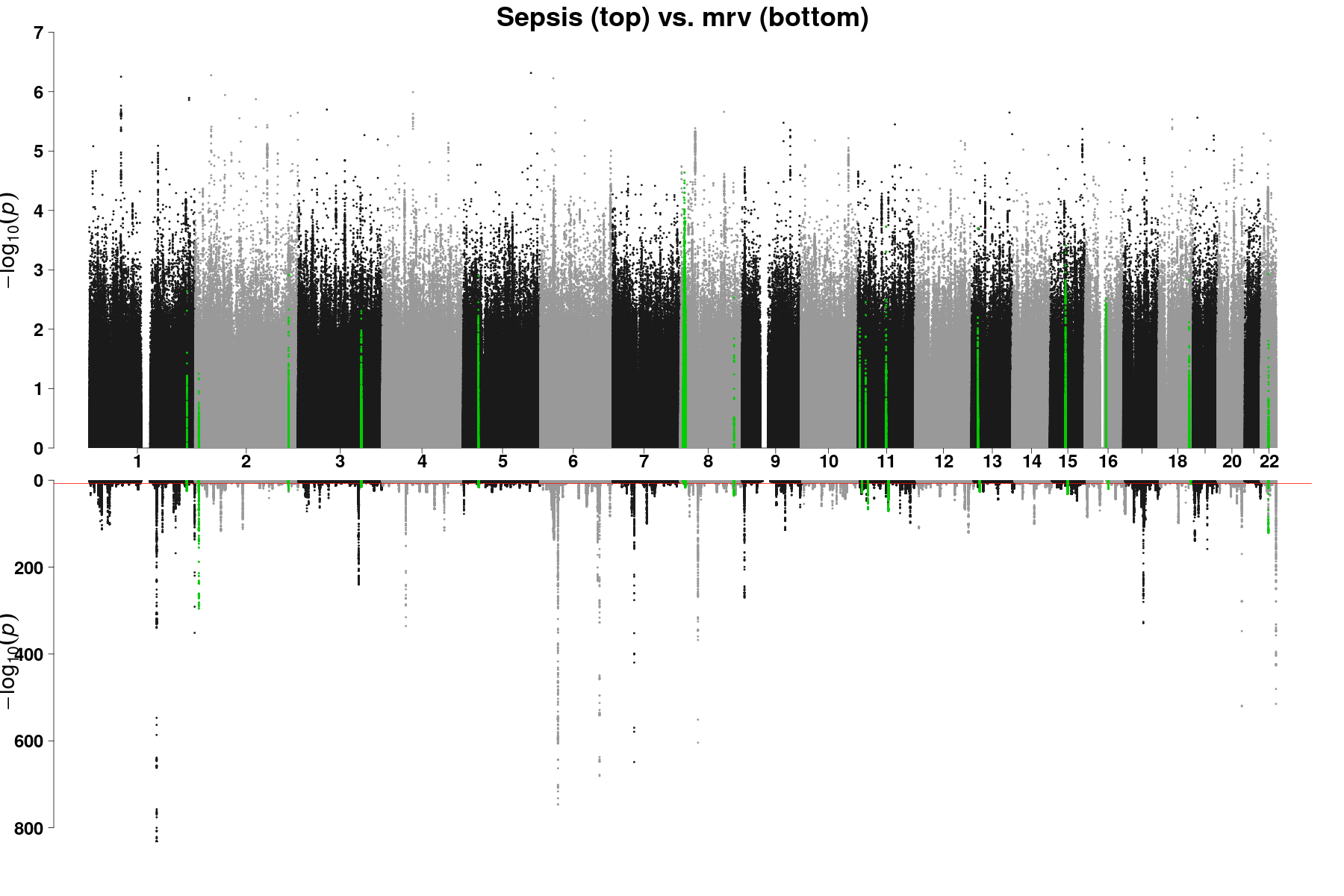


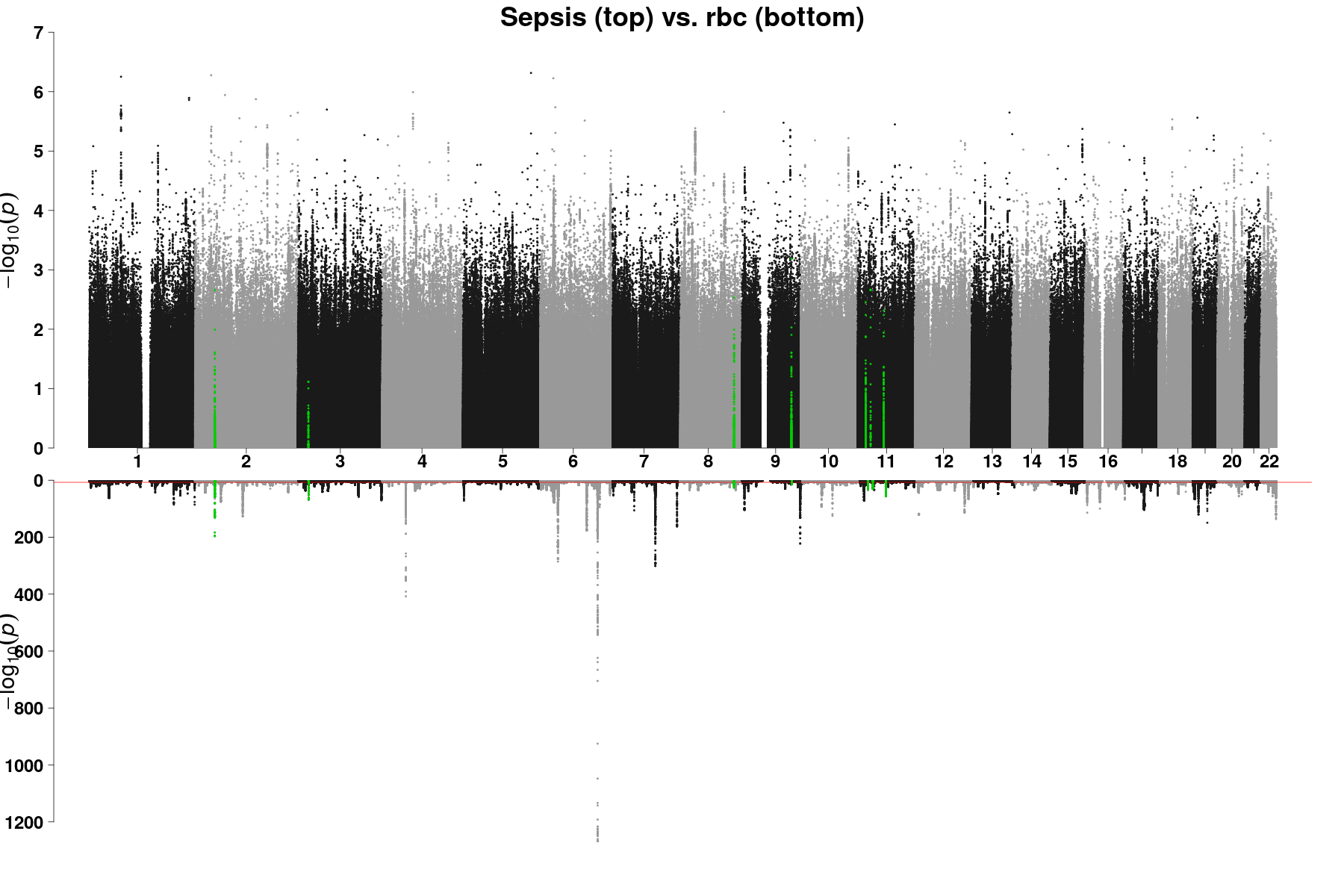

Supplement: S2 File — Local genetic correlation results for three additional red blood cell indices are visualized through mirrored Manhattan plots. There are three sub-figures, in the order of MCH, MRV, and RBC respectively. For each sub-figure, we show a mirrored Manhattan plot where the top panel shows the -log10(p-value) of sepsis GWAS on the top panel and the -log10(p-value) of the blood cell trait GWAS on the bottom panel. (DOCX) [file pone.0333675.s002.docx]
